# MLP-mmWP: High-Precision Millimeter Wave Positioning Based on MLP-Mixer Neural Networks

**DOI:** 10.3390/s23083864

**Published:** 2023-04-10

**Authors:** Yadan Zheng, Bin Huang, Zhiping Lu

**Affiliations:** 1 State Key Laboratory of Networking and Switching Technology, Beijing University of Posts and Telecommunications, Beijing 100876, China; 2 Hangzhou Innovation Institute, Beihang University, Hangzhou 310051, China; 3 State Key Laboratory of Wireless Mobile Communications, China Academy of Telecommunications Technology (CATT), Beijing 100876, China

**Keywords:** MMW communication, MMW positioning, MLP-Mixer neural network, beamforming fingerprints, deep learning

## Abstract

Millimeter wave (MMW) communication, noted for its merit of wide bandwidth and high-speed transmission, is also a competitive implementation of the Internet of Everything (IoE). In an always-connected world, mutual data transmission and localization are the primary issues, such as the application of MMW application in autonomous vehicles and intelligent robots. Recently, artificial intelligence technologies have been adopted for the issues in the MMW communication domain. In this paper, MLP-mmWP, a deep learning method, is proposed to localize the user with respect to MMW communication information. The proposed method employs seven sequences of beamformed fingerprints (BFFs) to estimate localization, which includes line-of-sight (LOS) and non-line-of-sight (NLOS) transmissions. As far as we know, MLP-mmWP is the first method to apply the MLP-Mixer neural network to the task of MMW positioning. Moreover, experimental results in a public dataset demonstrate that MLP-mmWP outperforms the existing state-of-the-art methods. Specifically, in a simulation area of 400 × 400 $m^{2}$, the positioning mean absolute error is 1.78 m, and the 95th percentile prediction error is 3.96 m, representing improvements of 11.8% and 8.2%, respectively.

## 1. Introduction

Millimeter wave (MMW) positioning is a promising technology based on 5G/6G communication systems [1,2,3]. Assuming that the system of MMW channels is modeled via deep learning techniques or other mathematical models, the location of user devices can be accurately inferred [4,5,6]. MMW localization may be used in indoor, outdoor, urban, or rural settings where large-scale 5G communication bases have been established and is not restricted to a single application scenario. In addition, the MMW positioning model relies on the channels alone through which a user device communicates with the bases [7]. Compared to a global navigation satellite system (GNSS), MMW localization is a passive process that can be embedded in 5G/6G communication systems [1]. Moreover, given its sufficiently wide bandwidth, high-speed transmission, and improved spectral efficiency, MMW technology is expected to attracted enormous attention for use in future communication systems due to its high-resolution sensing ability with centimeter-level localization accuracy [8,9,10].

Unfortunately, the propagation characteristics of MMW are subject to severe path loss with limited propagation distance. There is almost no LOS path between two points in MMW communications. Traditional MMW positioning only works effectively when there is an indoor or outdoor ultra-LOS pathway to the target. Massive multiple-input and multiple-output (MIMO) antennae merge beamforming (BF) technology in a system that broadcasts a steerable and directive radiation pattern to overcome these flaws [11,12]. Rich spatial information with several beamforming fingerprints (BFFs) may be produced by MMW transmission with BF technology. Fingerprinting solutions with deep learning methods based on MMW localization utilize sophisticated abilities of neural networks to build complex channel models of these BFFs in MIMO antenna systems [13,14]. Furthermore, regardless of whether there are LOS paths in these scenarios, these MMW localization neural network models learn the real-world structure information and the MMW propagation characteristics [15]. Therefore, the MMW fingerprinting positioning method can more effectively estimate a location based on an exact angle of arrival (AoA) than traditional communication carrier information, which increases the precision of base station positioning [16]. After being trained for a particular situation, the localization model may be used in complicated real-world scenes such as outdoor urban areas with non-line-of-sight (NLOS) routes. For the reasons listed above, MMW localization is an efficient way to compensate for the shortcomings of inaccurate GNSS positioning in indoor and outdoor urban areas.

Artificial intelligence technologies have recently boosted interest in the MMW location fingerprinting domain because of their powerful capabilities [17,18]. Convolutional neural networks (CNNs) and deep neural network modules are proposed in [19,20,21] that are devoted to precisely identifying the location and orientation of mobile devices. The authors of [22] used Kernel-based machine learning algorithms to forecast the positions of vehicles. An MMW positioning method combining Kalman filtering and machine learning was presented to forecast a robot’s static location [23]. A guide for MMW localization and user selection using a very large antenna array and model-based neural networks was developed in [24]. Using the Multi-Objective Generalized Normal Distribution Optimization algorithm, positions and ratings of devices can be determined to reduce fuel costs [25]. The prediction of the strongest path in the link state was examined with a machine-learning-trained classifier [26]. In [27,28,29], novel neural networks were used to improve positioning accuracy.

In contrast to the methods mentioned above, some researchers have used novel network models with beamformed fingerprints, which are multilayer perceptron mixer (MLP-Mixer) neural networks. MLP-Mixer uses numerous multilayer perceptrons (MLPs) to replace the convolution operation (Conv) in traditional CNNs and the self-attention mechanism in the transformer. Many scholars have employed this method to solve computer vision issues, as its linear timing sequence simplifies the operation structure and improves operational efficiency. We used this method to verify our novel deep learning structure, which is suitable for localization. In this study, a novel MLP-Mixer [30] architecture is proposed to achieve the accurate positioning of mobile devices, which depends on BFFs. Our contributions are summarized as follows:

- A fashionable and up-to-date AI concept MLP-Mixer named MLP-mmWP is first adopted and introduced for the task of MMW positioning;
- MLP-mmWP is an end-to-end neural network framework that does not rely on any handcrafted feature operation. It can predict the user location using communication information between the base and the user’s device;
- This study proves that MLP-Mixer is an effective method for the task of MMW positioning. Moreover, extensive experiments conducted in a popular public dataset (https://github.com/gante/mmWave-localization-learning, accessed on 10 April 2022) of outdoor scenarios demonstrate that MLP-mmWP can achieve high positioning accuracy with various noise levels and distinctly outperforms other the state-of-the-art (SOTA) methods.

The rest of this paper is organized as follows. Section 2 describes an MMW outdoor localization system. The detailed operation and the proposed method are presented in Section 3. In Section 4, the result of extensive experiments is elaborated and analyzed. Finally, conclusions are drawn in Section 5.

## 2. System Model

### 2.1. System Modeling

**Figure 1:**
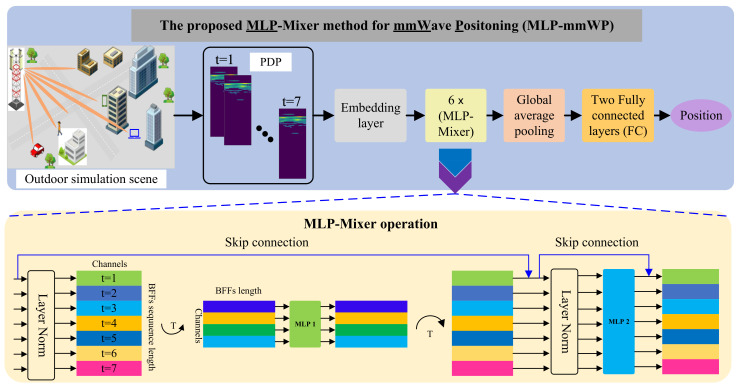
The pipeline of the MLP-mmWP method. The input data are the seven successive BFF data points (sampling in 7s), and the output is the predicted position ($x_{p},y_{p}$). The yellow box at the bottom illustrates the detailed architecture of the MLP-Mixer operation.

The MIMO system model of outdoor MMW localization is displayed in Figure 1. MMW power delay profile (PDP) data from a BS can be transmitted to a mobile device. A mobile device can receive MMW PDP data from a BS. The transmitter BF transmits constant radiation to the mobile device. The target devices are able to detect the transmitted radiation using the same detection method in order to obtain the required data.

In a MIMO MMW communication system, we consider a base station (BS) equipped with $N_{t}$ antennae that transmit directive and narrow beam patterns through a certain frequency band. A fixed code book is used to transmit BF vectors, where $C_{T}$ with a $B_{T}$ beam pattern covers all possible MMW transmission angles in a measured region. The code book is represented as $C_{T}=\left\{F_{1},F_{2},\ldots ,F_{B_{T}}\right\}$, where the current beam pattern is $F_{i}\in C_{T}$. There are $N_{R}$ equipped antennae in mobile devices. The received signal ($r_{i}\left(f\right)$) in the mobile device for the *i*th transmitted beam pattern in the frequency domain can be expressed as

(1) $$r_{i}\left(f\right)=wHF_{i}s+wz,i\in 1,2,\ldots ,K$$

where *w* is the BF vector in the mobile device; H denotes the channel matrix, which represents the transfer function of the MIMO system; $F_{i}$ represents the *i*th beam pattern; K is the size of the code book; *s* denotes the transmitted signal; and z corresponds to additional Gaussian white noise.

To gather the received power at all times, the data pattern of the receiving devices should be consistent with the transmitted data pattern, and those receivers must be synchronized with the BS. Consequently, the mobile device must utilize a fixed code book ($C_{R}$) with a $B_{R}$ beam pattern, where $C_{R}=\left\{W_{1},W_{2},\ldots ,W_{B_{R}}\right\}$. The design of a mobile device’s code book should cover all possible angles of arrival, and a similar gain should be required for these angles. Each transmitting beam pattern only saves the highest measured value in order to produce a BFF map. The saved data ($m_{i}\left[n\right]$) are defined as Equation (2).

(2) $$m_{i}\left[n\right]=\max_{p\in \left\{1,2,\ldots ,B_{R}\right\}}r_{i}^{p}\left(nT_{0}\right),n=1,2,\ldots ,N.$$

This equation presents the *n*-th sample from the *i*-th transmitting beam pattern, where $r_{i}^{p}\left(•\right)$ is the received signal of the *i*-th transmitted beam pattern and the *p*-th received BF pattern, and $T_{0}$ denotes the sample period. The received PDP values can be obtained from the data $m_{i}\left[n\right],n=1,2,\ldots ,N$, where *N* is the number of samples. Consequently, a two-dimensional BF image map can be constructed using the received PDP with all the transmitted beam patterns ($B_{T}$). The pixel (*M*) of this map depicts the all PDP. Thus, the two-dimensional BF image map (*M*) can be established using the received PDP, which can be expressed as:

(3) $$M=\left[\begin{array}{cccc} m_{11} & m_{12} & \cdots & m_{1K}\\ m_{21} & m_{22} & \cdots & m_{2K}\\ \vdots & \vdots & \ddots & \vdots \\ m_{N1} & m_{N2} & \cdots & m_{NK} \end{array}\right].$$

where *K* is less than or equal to $B_{T}$.

### 2.2. Problem Definition

**Figure 2:**
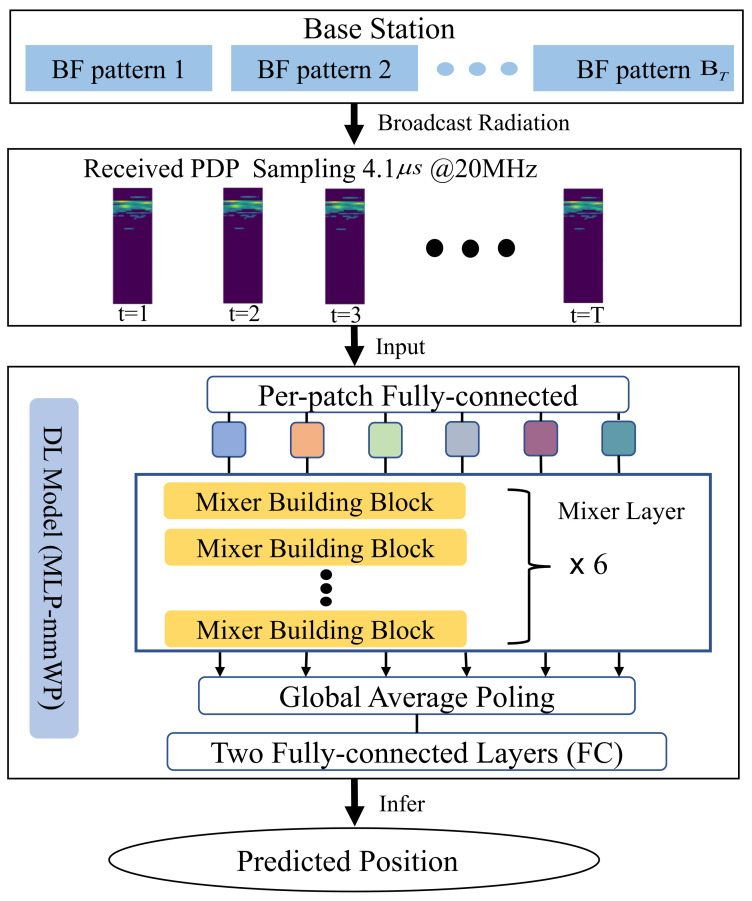
The overall scheme of system modeling and the problem definition of MMW positioning.

As depicted in Figure 2, the received PDP with various of BF patterns is sampled at 20MHz over a span of 4.1 μs. The successive received PDP samples are generated in each mobile device once per second for 13 s. Then, the successive PDPs are fed into the deep learning (DL) model to train/infer the current position of the mobile devices. Pedestrians and vehicles are randomly distributed throughout the New York University (NYU) area; the movement parameters are listed in Table 1.

### 2.3. Simulation Setting

In this study, an open-source 3D map of the NYU area is used to build BFF data, comprising a 400 × 400$\;m^{2}$ area. The Wireless InSite ray-tracing simulator (https://www.remcom.com/wireless-insite-em-propagation-software/, accessed on 10 April 2022) is applied to simulate the MMW BF patterns. In this simulation system, the carrier frequency is fixed as 28 GHz; TX transmit power is set to 45 dBm; the codebook size is K=32, which covers a 155 arc with 5 between each BF pattern; and the size of receiver grid is 1 m (1 m above the ground).

As in [15], the received power data sampling rate is 20 MHz for 4.1 μs. Thus, the number of sample TX BFs is theoretically 82, while the number of real-world samples is N=81 in the published data (https://github.com/gante/MMW-localization-learning, accessed on 20 March 2022). Therefore, for K=32 BF patterns, the dimension of *M*, defined as Equation (3), is $R^{\left[32,81\right]}$ in each sampling moment.

## 3. Methods

According to experimental results reported in the literature [31], the most appropriate number of input BFF sequences is seven because longer sequences are not able to improve the accuracy of the predicted position, and shorter sequences will degrade the performance. Thus, sequences of seven BFFs are also employed to estimate the positions of mobile devices inthis study.

### 3.1. Background of MLP-Mixer

MLP-Mixer [30], which is used for image classification, is a deep neural network entirely depending on multilayer perceptrons (MLPs) first proposed in 2021. MLP-Mixer, which is based on ViT, is a novel and effective deep leaning framework for computer vision. In contrast to LSTM, MLP-Mixer, with two MLPs, is designed as a parallel processor to communicate channel and spatial (or time dimension) information [32]. Furthermore, MLP-Mixer has been proven to effectively process temporal signals. Inspired by this performance, a novel deep learning model based on MLP-Mixer is proposed to estimate the position of mobile devices using a sequence of BFFs. The MLP-Mixer operation is presented in Figure 1 (the pale yellow box), and the detailed operations can be expressed as Equations (4)–(6).

(4) $$Y={\mathrm{MLP}}_{1}\left(F_{i}^{T}\right),$$

(5) $$Y_{s}=F_{i}+Y^{T},$$

(6) $$F_{o}=Y_{s}+{\mathrm{MLP}}_{2}\left(Y_{s}\right),$$

where $F_{i}$ is the input feature map; $F_{i}^{T}$ and $Y^{T}$ denote the transposition of $F_{i}$ and *Y*, respectively; $Y_{s}$ denotes the output result of the skip-connection operation; ${\mathrm{MLP}}_{1}$ and ${\mathrm{MLP}}_{2}$ represent the distinct MLP operations; and $F_{o}$ is the output feature map of the MLP-Mixer operation.

### 3.2. The Proposed Method and Training Details

**Data preprocessing.** The detailed architecture of MLP-mmWP is illustrated in Figure 1. The input is seven successive BFF data point that are beamformed from BS with a mobile communication device. Thus, the original dimension of the input is [7,32,81], which is then is reshaped to $I\in R^{\left[7,2592\right]}$, which is the actual input dimension of MLP-mmWP.

**Embedding layer.** The embedding layer is a 1D convolutional neural network, and the subsequent activation function is ReLU. The kernel size and stride of the convolutional operation are both set to 1, and the output dimension is 1024. The embedding layer can be mathematically expressed as:

(7) $$E_{o}=Embedding\left(I,w_{E}\right),$$

where $E_{0}\in R^{\left[7,1024\right]}$ is the output of the embedding layer, and $w_{E}$ is the parameter of the convolutional operation for embedded decoding.

**MLP-Mixer feature extraction.** In this study, 6 MLP-mixer operations are employed in the MLP-MWP pipeline. The MLP-Mixer operation is represented as in Equations (4)–(6), and the dimension of each MLP-Mixer operation does not change. The output of the MLP-Mixer module can be regarded as the deeper feature map. The computing process can be expressed as:

(8) $$M_{o}=MLPMixer\left(E_{o},w_{M}^{i}\right)$$

where $M_{o}\in R^{\left[7,1024\right]}$ is the output of six MLP-Mixer layers, and $w_{M}^{i}\left(i=1,2,\ldots ,6\right)$ represents the parameters of the *i*th layer of the MLP-Mixer module.

**Position estimator.** As shown in Figure 1, the position estimator includes one global average pooling layer and two fully connected (FC) layers.

(9) $$G_{o}=GP\left(M_{o}\right)$$

(10) $$\left(x_{p},y_{p}\right)=FC\left(G_{o},w_{F}^{j}\right)$$

where GP and FC denote the global average pooling and FC operation, respectively; $w_{F}^{j}\left(j=1,2\right)$ is the parameter of the two FC layers; and $x_{p}$ and $y_{p}$ represent the x and y coordinate of the predicted location, respectively.

**Training details.** The mean square error loss function is employed to optimize the MLP-mmWP neural network, which can be expressed as Equation (11) for each epoch.

(11) $$L=\frac{1}{N}\sum_{n=1}^{N}\left({\left(x_{p}^{n}-x_{r}^{n}\right)}^{2}+{\left(y_{p}^{n}-y_{r}^{n}\right)}^{2}\right)$$

where N=128 denotes batch size in the training phase, and $x_{r}$ and $y_{r}$ represent the x and y coordinate of the real location, respectively.

Adam optimizer and Cosine learning rate decay with warmup are used in the training phase. The proposed method trains on a single GPU (TITAN RTX 24G) with a batch size of 128 and runs for 300 epochs.

## 4. Experiments and Results

As in [31], the mean absolute error (MAE) and its 95% percentile serve as the main metrics. MAE is the mean Euclidean distance between the predicted location ($\left(x_{p},y_{p}\right)$) and the reference location ($\left(x_{r},y_{r}\right)$) on all testing samples distributed in a $400\times 400\;m^{2}$ area. To demonstrate the robustness and effectiveness of the proposed method (MLP-mmWP), various noise levels (σ = 2, 4, 6, 10 dB (Log-Normal)) are introduced into BFFs. The quantitative performance of MLP-mmWP with the input BFFs and various noise levels is presented in Table 2. The results demonstrate that MLP-mmWP can robustly estimate the location of a mobile device even under some levels of noise.

**Figure 3:**
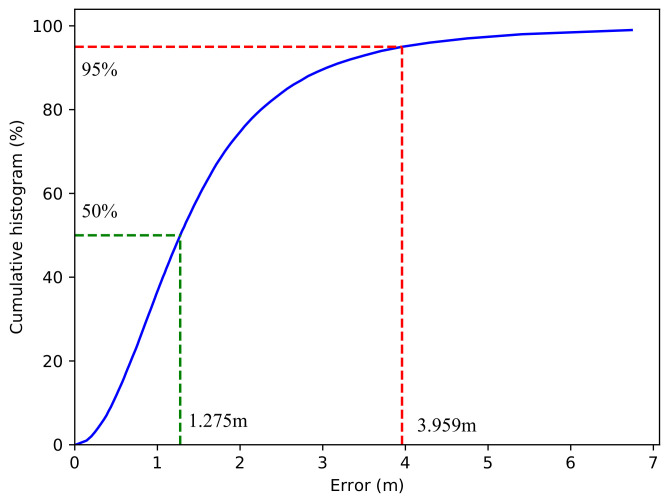
The error-cumulative histogram of the proposed MLP-mmWP method (employing sequences of seven BFFs and with a noise level of σ=6 dB). The green dotted line is the median error and the red dotted line represents the 95th percentile error.

To achieve a comprehensive comparison with the method proposed in [31], the error-cumulative histogram of MLP-mmWP with a noise level of σ=6 dB is illustrated in Figure 3. The predicted errors of 50% are within 1.275 m (improved by 22.3% relative to the TCN method (1.641 m)), and the confidence level within 3.959 m is 95% (improved by 18.5% relative to the TCN method (4.693 m) [31]).

The results of comparisons with other state-of-the-art methods are depicted in Table 3. The testing results are computed with BFFs of σ=2 dB, which show that MLP-mmWP achieves far better results than positioning estimation via other methods. Even compared to the suboptimal method, the performance of MLP-mmWP is improved by 11.8% and 8.2% in terms of the metrics of MAE and 95th percentile, respectively. Therefore, the proposed method further improves the positioning accuracy and the localization error based on MMW communications close to the meter level.

**Figure 4:**
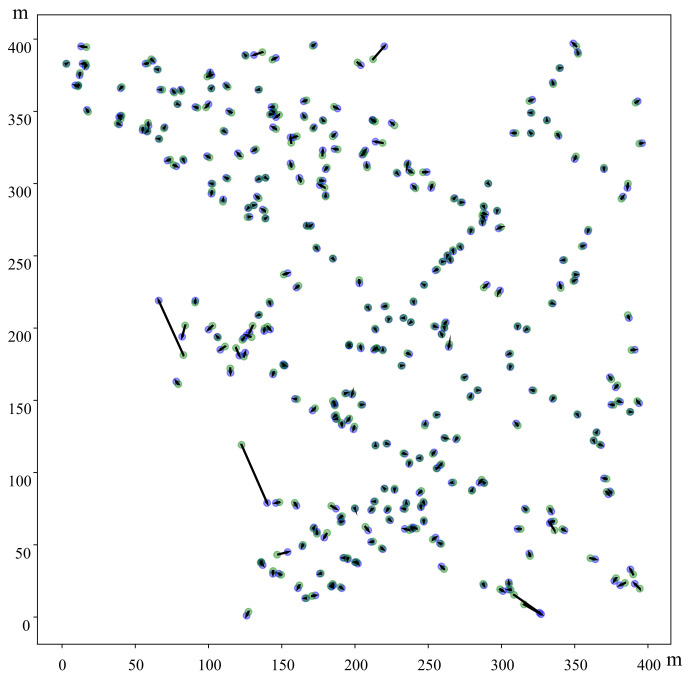
Error visualization of 300 randomized testing samples. The green points represent the reference locations, and the blue points denote the locations predicted by the proposed method. The black lines represent the deviation between the reference locations and predicted locations.

Finally, the visualized differences between the positions estimated by MLP-mmWP and the real positions of mobile devices are depicted in Figure 4. The length of each vector denotes the Euclidean distance between the predicted location and the real location. Figure 4 shows that MLP-mmWP achieves high positioning accuracy in the 400 × 400 $m^{2}$ area. Only three predicted positions of mobile devices are unacceptable among the 300 visualized samples randomized from testing data.

## 5. Discussion

The simulation data demonstrate that the proposed method is feasible to localize the position of a user. We also recognize that there are some challenges for directly applying the model parameters in real-world communication systems because the real physical world is more complicated than the simulation scenario. The retrained or fine-tuned the deep learning model could be applied to a real-world scenario if real-world communication information could be accessed. Therefore, the primary challenge of implementing this method is to access large amounts of real-world communication data that contain a large scale of testing points covering many typical scenarios.

## 6. Conclusions

Based on the vast MMW spectrum and narrow-beam antenna technology, in this study, we explored the implementation of a precise positioning method in 5G mobile communication systems. Inspired by the excellent performance of MLP-mixer in processing temporal signals, we proposed a novel deep learning method named MLP-mmWP to estimate the localization of mobile users of 5G communications. MLP-mmWP can achieve a mean absolute positioning error within 1.8 meters in an outdoor area of 400 × 400 $m^{2}$, even if the beamformed fingerprints are embedded in 2–10 dB of noise. In conclusion, MLP-mmWP distinctly improves the positioning accuracy and the localization error based on MMW communications near the meter level. Thus, the proposed method can be used to further real-world applications of millimeter wave positioning technology.

## Figures and Tables

**Table 1:** The parameters of our simulation environment.

Parameter Name	Pedestrian Parameters	Vehicle Parameters
Average speed	1.4 m/s	8.3 m/s
Max speed	2.0 m/s	13.9 m/s
Max acceleration	0.3 m/s${}^{2}$	3 m/s${}^{2}$
Max direction change	10	5
Probability of change	[0.8, 0.1, 0.05, 0.05]	[0.8, 0.02, 0.05, 0.13]

**Table 2:** Performance of the MLP-mmWP method with different levels of noise.

Noise Level (σ)	MAE (m)	95th Percentile (m)	50th Percentile (m)
2 dB	1.568	3.793	1.126
4 dB	1.660	3.898	1.219
6 dB	1.708	3.959	1.275
10 dB	1.772	4.038	1.411

**Table 3:** Results of comparisons with other state-of-the-art methods. The best performance is labeled in boldface, and the suboptimal performance is underlined.

Method (Year)	MAE (m) ↓	95th Percentile (m) ↓
[15]-TCN (2020)	2.03	5.81
[31]-CNN (2020)	5.38	13.66
[31]-LSTM (2020)	2.09	5.06
[31]-TCN (2020)	1.78	4.13
[13] (2021)	2.56	7.02
This work (MLP-mmWP)	1.57 (11.8%)	3.79 (8.2%)

## Data Availability

Not applicable.
